# Impact of Aging and Exercise on Mitochondrial Quality Control in Skeletal Muscle

**DOI:** 10.1155/2017/3165396

**Published:** 2017-06-01

**Authors:** Yuho Kim, Matthew Triolo, David A. Hood

**Affiliations:** ^1^Muscle Health Research Centre, York University, Toronto, ON, Canada M3J 1P3; ^2^School of Kinesiology and Health Science, York University, Toronto, ON, Canada M3J 1P3

## Abstract

Mitochondria are characterized by its pivotal roles in managing energy production, reactive oxygen species, and calcium, whose aging-related structural and functional deteriorations are observed in aging muscle. Although it is still unclear how aging alters mitochondrial quality and quantity in skeletal muscle, dysregulation of mitochondrial biogenesis and dynamic controls has been suggested as key players for that. In this paper, we summarize current understandings on how aging regulates muscle mitochondrial biogenesis, while focusing on transcriptional regulations including PGC-1*α*, AMPK, p53, mtDNA, and Tfam. Further, we review current findings on the muscle mitochondrial dynamic systems in aging muscle: fusion/fission, autophagy/mitophagy, and protein import. Next, we also discuss how endurance and resistance exercises impact on the mitochondrial quality controls in aging muscle, suggesting possible effective exercise strategies to improve/maintain mitochondrial health.

## 1. Introduction

Skeletal muscle accounts for approximately 40% of total body mass, and it plays an indispensable role in locomotion and metabolism. Skeletal muscle undergoes a gradual loss of fat-free mass, size, and function in the aging process, called *sarcopenia* [1]. The etiology of sarcopenia is complex and involves the interplay of various factors such as oxidative stress, physical inactivity, imbalanced protein homeostasis, apoptosis, inflammation, malnutrition, and/or mitochondrial dysregulation [2–5]. Mitochondria play an essential role in the aging-related muscle deterioration because of their importance in the production of energy and reactive oxygen species (ROS) [6], apoptotic signaling, and calcium (Ca^2+^) handling [7]. Thus, the natural aging process, along with coincident inactivity, progressively impairs mitochondrial integrity which might be a leading factor for sarcopenia.

The underlying mechanisms of aging-associated mitochondrial dysregulation in skeletal muscle remain incompletely understood. Morphologically, aging muscles appear to have either fragmented, round-shaped mitochondrial networks [8] or unusually enlarged mitochondrial fragments [9, 10]. For example, subsarcolemmal (SS) and intermyofibrillar (IMF) mitochondrial fractions in the skeletal muscle of old rats tend to be thinner and smaller, respectively [8]. In contrast, other findings have shown that aging muscle mitochondria appear as an elongated, “giant” network [9, 10]. To better understand these inconsistent findings in the mitochondrial structure of aging muscle, it becomes important to investigate the mechanisms involved in mitochondrial turnover, the balance between organelle biogenesis, dynamics, and degradation, which may also help delineate the underlying causes of aging-related dysregulation of mitochondria in skeletal muscle.

Exercise and physical activity have been suggested as effective tools for either improving the quality of aging muscle or delaying the onset of sarcopenia, yet the underlying mechanisms in the exercise-inducible adaptations are still obscure. A growing number of studies have sought to define mitochondrial adaptations in aging skeletal muscle following various exercise regimens. While mitochondrial biogenesis has been relatively well investigated, research interests have more recently been directed to other mitochondrial dynamic mechanisms (fusion/fission; autophagy/mitophagy) in aging muscle.

This review summarizes current findings on the aging-related mitochondrial adaptations in skeletal muscle, with a specific focus on mitochondrial biogenesis and dynamic controls. In addition, this paper also outlines current research findings on the effects of exercise on mitochondrial quality control in aging skeletal muscle.

## 2. Mitochondrial Biogenesis and Aging

**Figure 1:**
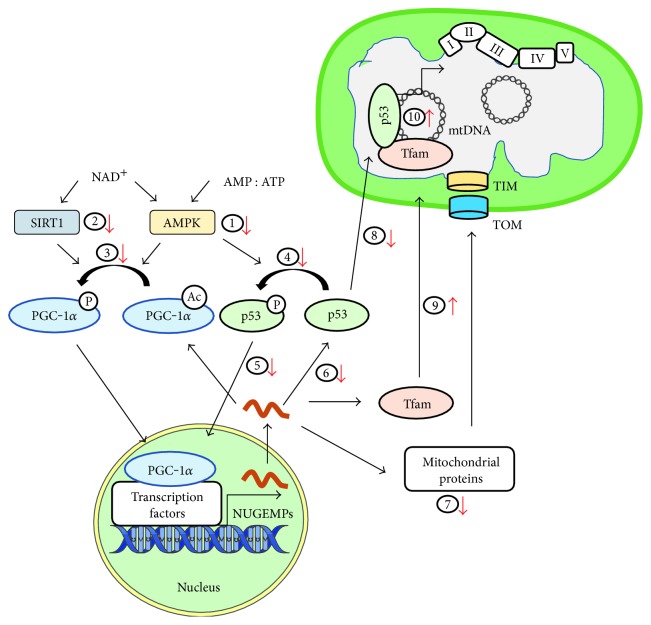
Aging is associated with reductions in mitochondrial biogenesis. Initial signaling through (1) AMPK and (2) SIRT1 is reduced with aging, thereby reducing (3) PGC-1*α* coactivation and (4) p53 activation of (5) NUGEMP expression, leading to a decrease in (6) PGC-1*α* protein and (7) mitochondrial targeted proteins. However, aging is associated with increased (8) TFAM and (9) p53 which has the capacity to enhance (10) mtDNA replication. Depending on age, this mtDNA may contain elevated mutations and may not promote efficient biogenesis in skeletal muscle.

The synthesis of new mitochondria, termed mitochondrial biogenesis, promotes the expansion of an existing mitochondrial network. This process is constantly ongoing within skeletal muscle in order to maintain mitochondrial content and function in response to various stimuli including exercise, as well as other cellular stressors. Aging is known to be a leading factor for the reductions in mitochondrial components and capacity [11–13]. Furthermore, aged skeletal muscle has a reduced ability to synthesize mitochondria in response to biogenesis-inducing factors [14, 15]. Thus, an understanding of the regulation of mitochondrial biogenesis in healthy skeletal muscle, and the changes associated with advanced aging, is important in developing an intervention to prevent the progression of sarcopenia (Figure 1).

### 2.1. PGC-1*α* as a Regulator of Mitochondrial Biogenesis

Mitochondrial biogenesis requires the coordination of the nuclear and mitochondrial genomes, as 99% of approximately 1150 mitochondrial proteins are nuclear-encoded [16], whereas only 13 proteins, along with 2 rRNAs and 22 tRNAs, are mitochondrially encoded [17, 18]. Peroxisome proliferator-activated receptor (PPAR) gamma coactivator 1*α* (PGC-1*α*) is a master regulator of this process [19, 20], and it plays a significant role in muscular phenotypic changes and aerobic performance. Studies utilizing overexpression [19, 21, 22] and deletion [23–25] of PGC-1*α* have shown that it is critical in determining the oxidative phenotype and mitochondrial content in skeletal muscle. Functionally, PGC-1*α* coactivates various transcription factors, such as nuclear respiratory factors 1 and 2 (NRF-1/2), PPAR*γ*, and estrogen-related receptors (ERR), all of which are important in activating the expression of nuclear genes encoding mitochondrial proteins (NUGEMPs) [26–29]. A critical PGC-1*α*-regulated NUGEMP is a mitochondrial DNA-specific transcription factor, transcription factor A of the mitochondria (Tfam), which serves to coordinate the nuclear and mitochondrial genomes in the regulation of mitochondrial biogenesis [30]. Furthermore, PGC-1*α* coactivates its own gene expression by positive feedback, thus inadvertently acting to increase its protein content as well [31, 32].

Various splice variants of PGC-1*α* have been identified within skeletal muscle. For example, the full-length isoforms, PGC-1*α*1–3, are associated with mitochondrial biogenesis and oxidative phosphorylation [33], and the truncated variants, NT-PGC-1*α*, are produced by alternative 3′ splicing of PGC-1*α* mRNA at exon1a. These truncated variants are expressed in a similar ratio to that of PGC-1*α* in skeletal muscle [34]. In contrast, a truncated splice variant of PGC-1*α*, termed NT-PGC-1*α*-b or PGC-1*α*4, is involved in muscle hypertrophy [35, 36]. Although several studies have indicated that these truncated variants are upregulated by cold exposure in brown adipose tissue [37] and are differentially regulated by various exercise intensities in skeletal muscle [38], the underlying mechanisms of actions of these variants within skeletal muscle are not well understood, and even less studied in the context of aging. A variety of studies have indicated that PGC-1*α* is responsive to stimuli such as Ca^2+^ [31, 39–41], ROS [42], nitric oxide [43], thyroid hormone [44, 45], and increased energy imbalances such as nutrient deprivation [25, 46, 47] and exercise [44, 48]. Thus, alterations in signaling from these sources can lead to changes in mitochondrial content within skeletal muscle, which are also linked with aging-associated alterations in PGC-1*α* expression.

Considering the importance of PGC-1*α* in maintaining skeletal muscle mitochondrial content through organelle biogenesis, aging-associated modifications in the expression and/or activation of PGC-1*α* are a timely and highly relevant research area. Although contradictory findings were observed in human studies [11, 49, 50], aging is associated with a decline in PGC-1*α* expression in the skeletal muscle of rodents [51]. The age-related reductions in PGC-1*α* have the potential to reduce the transcriptional drive for mitochondrial biogenesis, partially explaining the decreased skeletal muscle mitochondrial content associated with age. Further, knockdown of PGC-1*α* expression in mice intensifies the decline in mitochondrial gene expression and function in aging skeletal muscle [52].

PGC-1*α* is also important for preventing or delaying the onset of muscle atrophy by suppressing atrophy-related gene expression, through the inhibition of Forkhead box O3a (FoxO3a), a potent transcriptional inducer of muscle atrophy. For example, PGC-1*α* overexpression in adult rodents suppressed FoxO3a activity, promoted muscle mass maintenance [53], and similarly prevented starvation-associated protein degradation and atrophy in myotubes [54]. Together, this may explain the contribution of age-related PGC-1*α* deficits to the phenotypic loss of muscle size, which thus suggests the therapeutic potential of this protein in the prevention of sarcopenia.

### 2.2. AMPK and NAD^+^

Various regulatory networks converge to activate PGC-1*α* and promote mitochondrial biogenesis. In response to a reduced cellular energy status, energy-sensing networks associated with AMP-activated protein kinase (AMPK) and silent mating type information regulation 2 homolog 1 (SIRT1) are activated via AMP and nicotinamide adenine dinucleotide (NAD^+^), respectively. These proteins converge at PGC-1*α* to promote organelle biogenesis [55, 56]. AMPK is an energy-sensitive kinase that is activated by low energy status, as signified by an increase in the AMP : ATP ratio [57–59], and it also phosphorylates PGC-1*α* on threonine-177 and serine-538 [44, 60–62]. SIRT1 is an NAD^+^-dependent deacetylase that acts on PGC-1*α* in response to an increase in NAD^+^, which is also indicative of a reduction in cellular energy [32, 46, 63, 64]. AMPK and SIRT1 are functionally interdependent, as AMPK can increase NAD^+^ and subsequently activate SIRT1 within muscle, and vice versa [55, 57, 65]. Moreover, AMPK may function as a switch between PGC-1*α*-dependent and PGC-1*α*-independent mitochondrial biogenesis pathways which are promoted by SIRT1 [66]. Nevertheless, the phosphorylation and subsequent deacetylation of PGC-1*α* by AMPK and SIRT1 activate PGC-1*α* and promote mitochondrial biogenesis. Using exercise, caloric restriction, and/or pharmacological activation models, it has been shown that both AMPK and SIRT1 promote an oxidative phenotype within skeletal muscle, along with increased mitochondrial content [44, 46, 56, 58, 64, 65, 67–71].

Most reports on aged skeletal muscle show blunted AMPK activation in response to exercise and AICAR treatment [14, 70], with no apparent change in AMPK expression [72]. These findings may partially explain the age-associated declines in mitochondrial biogenesis since diminished AMPK activation may downregulate PGC-1*α*. It may further indicate a mechanism whereby reductions in AMPK activity in aging muscle may reduce its ability to increase NAD^+^ levels and activate SIRT1. This evidence suggests that targeting AMPK activation within skeletal muscle may promote mitochondrial biogenesis and thus healthier skeletal muscle with age.

Reductions in NAD^+^ are evident within aged skeletal muscle due to an increase in its breakdown, without reductions in SIRT1 protein [66, 73, 74], suggesting that this signaling pattern toward mitochondrial biogenesis may be hindered with aging. Using a knockdown model for cell-specific nicotinamide mononucleotide (NMN) adenylyltransferase (NMNAT) which regulates NAD^+^ levels within skeletal muscle, it was found that reductions in nuclear NAD^+^ are partially responsible for the deficits in oxidative phosphorylation (OXPHOS) and mitochondrial biogenesis [66], which may help to explain the reductions in mitochondrial content with age. In the same study, aging mice treated with the NAD^+^ precursor, NMN, restored skeletal muscle NAD^+^, as well as increased mitochondrial function and OXPHOS gene expression [66]. In addition, nicotinamide ribose treatment was also shown to increase NAD^+^ and to prevent age-related muscle stem cell senescence, along with an improvement in mitochondrial and muscle health with age [75]. These data support the idea that age-related deficits in NAD^+^ are partially responsible for the age-associated reduction in mitochondrial biogenesis and suggest a possible target for the replenishment in aging muscle.

### 2.3. Mitochondrial DNA

A unique characteristic of the mitochondrion is that it possesses its own mitochondrial DNA (mtDNA), a circular molecule of approximately 16.6 kb in size. mtDNA exists as a result of the mitochondrial evolutionary upbringing as a prokaryote, maintaining a symbiotic relationship with eukaryotic cells [76, 77]. Within each organelle, there are multiple maternally inherited copies of mtDNA, which are heteroplasmic due to the variant nature of mtDNA copies within the cell [78].

Mitochondrial gene transcription is initiated by the nuclear-encoded mitochondrial RNA polymerase (POLRMT) [79], with the aid of mitochondrial transcription factors B1 and B2 (TFB1/2) and Tfam [80, 81]. Since the synthesis of new mitochondria depends upon the coordination of the nuclear and mitochondrial genomes, the integrity of mtDNA and the machinery involved in its replication and gene transcription are crucial in maintaining a healthy pool of mitochondria and efficient biogenesis.

Within the skeletal muscle of humans, primates, and rodents, aging has long been associated with an accumulation of large-scale mtDNA deletions and mutations, which ultimately contribute to sarcopenia and age-related myopathies [3, 82–88]. This is likely because mtDNA is more readily exposed to damaging free radical species due to its close proximity to the electron transport chain (ETC), along with the lack of protective proteins that nuclear DNA possess [89]. Further, replicative damage to mtDNA leads to elevated ROS production [90, 91], which is seen in aged skeletal muscle [83], suggesting that aging is likely to lead to an impairment in mtDNA replication. Taken together, mtDNA damage likely ultimately reduces the quality and quantity of mitochondrial biogenesis in aging muscle.

Aging skeletal muscle has also been associated with declines in mtDNA copy number. For example, aged rats (27 months old) were shown to have 20–40% less mtDNA in skeletal muscle compared to young rats (6 months old), corresponding to reductions in mitochondrial transcripts in less oxidative muscle fibers [92]. However, it is unclear whether aging-related reductions in mtDNA copy number occur in humans, since inconsistent results have been reported, whereby no changes [93] and even increase in mtDNA have been observed [94]. Furthermore, elimination of mtDNA in cells altered nuclear gene expression and reduced mitochondrial proliferation [95], indicating that mtDNA is required for organelle biogenesis and nuclear coordination. These findings provide the opportunity to formulate two potential theoretical frameworks. If mtDNA reductions are evident in aged skeletal muscle, it may limit the potential for mitochondrial biogenesis, as alluded to above. On the other hand, if mtDNA is accumulating within aged skeletal muscle, it could be indicative of a compensatory mechanism in response to reduced respiratory chain function. This elevated level of mtDNA could contain mutations, thus resulting in further overall respiratory chain defects.

### 2.4. Transcription Factor p53

As a tumor suppressor, the transcription factor p53 promotes the expression of various genes involved in cellular defense systems such as apoptosis and cell cycle arrest in response to DNA damage [96, 97]. More recently, p53 has been identified as a regulator of mitochondrial integrity, content, and function as well as organelle biogenesis [98–103]. In particular, p53 can be colocalized within cytoplasm, nucleus, and mitochondria, whereby it facilitates both nuclear and mitochondrial gene expression [101, 104–106]. Mitochondrial p53 interacts with and stabilizes mtDNA [102], and mtDNA expression is dependent, in part, on the presence of p53 in skeletal muscle [107]. Indeed, the value of p53 to the mitochondrial genome became more evident following a study showing that an exercise-induced upregulation of p53 lessens mtDNA damage in the skeletal muscle of a mtDNA mutator mouse model of aging, compared to the mutator mouse model with skeletal muscle-specific knockdown of p53 [108].

Reductions in muscle mitochondrial content and complex assembly in p53 knockout (KO) mice further implicate p53 as an important factor for the maintenance of mitochondrial aerobic capacity [100, 109]. Within the nuclear genome, p53 supports mitochondrial biogenesis by upregulating the expression of genes indicative of oxidative phenotypes, such as Tfam and NRF-1, as well as the ETC assembly protein synthesis of cytochrome c oxidase (COX) 2 [23, 103, 105, 107, 110]. Moreover, PGC-1*α* has a p53 binding site in its promoter region [61, 111] so that p53 could potentially increase PGC-1*α* transcription, inducing downstream NUGEMP expression [112], further suggesting a role for p53 in the regulation of mitochondrial biogenesis. Within the mitochondrial genome, p53 induces the transcription of 16S rRNA and COX subunit I [101, 105]. Thus, the reductions in mitochondrial content in p53-KO animals are likely due to decreased p53-induced gene expression important to mitochondrial biogenesis.

Aging is associated with increases in skeletal muscle p53 protein, suggesting that p53 may promote a proapoptotic environment in aged muscle [113–116]. This increase is clearly insufficient to maintain mitochondrial content at levels similar to those observed in the muscle of young animals. p53 receives a regulatory input from AMPK, whereby AMPK phosphorylates and activates p53 [117–119]. Thus, the age-related deficiency in AMPK activation in rodent skeletal muscle (see above) potentially suppresses p53 activation and signaling for mitochondrial biogenesis. Contrary to AMPK, SIRT1 normally deacetylates and inactivates p53, whereby it is liberated from its stimulating effect on biogenesis [120, 121]. However, aging is associated with reductions in SIRT1 activity, which may promote the proapoptotic functions of p53 rather than biogenesis. Importantly, the aging-related reductions in PGC-1*α* may also reduce p53 because of its coactivity with PGC-1*α* [112]. It was recently revealed that aging is also correlated with reduced s-nitrosylation of p53, a modification that enhances p53 binding to the PGC-1*α* promoter and promotes its associated antioxidant response [122], further suggesting a reduced ability of p53 to promote biogenesis through its cooperative action with PGC-1*α*. However, more research is necessary to characterize the role of p53 in aged skeletal muscle and to determine if there is a therapeutic potential in targeting p53.

### 2.5. Mitochondrial Transcription Factor A (Tfam)

Regulation of mtDNA transcription and replication is mediated by factors such as POLRMT, mitochondrial TFB1M/TFB2M, and Tfam [80, 123], all of which are nuclear gene products. The transcription of Tfam, TFB1M, and TFB2M is activated by NRF-1 and NRF-2, which are, in turn, coactivated by PGC-1*α*, thus connecting the nuclear and mitochondrial genomes in mitochondrial biogenesis [30, 124].

Tfam is crucial for the regulation of mtDNA, and whole-body loss of its function is associated with embryonic lethality, whereas partial loss leads to reductions in mtDNA content and tissue-wide respiratory deficits [125, 126]. Categorically, Tfam has the high-mobility group box domains, which have the ability to induce a U-turn-like conformation of mtDNA [127–129]. Once this is completed, TFB2M and POLRMT are recruited to the H and L promoters of mtDNA allowing for gene transcription. Furthermore, Tfam packages and compacts mtDNA into nucleoid-like structures [130, 131], protecting this genome against ROS-induced mutations.

Tfam levels are positively correlated with mtDNA content [132, 133]. Within developing skeletal muscle, the increase in Tfam mRNA is associated with elevations in mitochondrial content and localization [134]. Mitochondrial biogenesis is associated with an elevated abundance in Tfam transcripts, as well as its mitochondrial localization [135–140], whereas muscle-specific depletion of Tfam serves to reduce mtDNA abundance [141]. Together, these data indicate an essential role for Tfam in promoting mtDNA replication, transcription, and its subsequent effects on elevating the synthesis of mitochondria.

Several studies suggest that Tfam is elevated in aged skeletal muscle, although reductions in mitochondrial content are evident. This was shown to be the case in the skeletal muscle of both aged rats [88] and humans [142]. In humans, these increases were correlated with increases in NRF-1 mRNA and protein bound to nuclear DNA [142]. Altogether, these data suggest that aging may lead to a compensatory increase in Tfam, probably to maintain or increase mitochondrial content and respiratory function. Nevertheless, it may also further promote the production of mutated mtDNA, leading to mitochondrial dysfunction.

## 3. Mitochondrial Dynamics and Aging

In addition to organelle biogenesis discussed in the previous sections, mitochondrial quality is finely adjusted by reshaping mitochondrial structures that are primarily controlled by fusion/fission, as well as autophagy/mitophagy. The following sections summarize our current understanding in these mitochondrial regulatory systems in skeletal muscle in the context of aging, as well as the mechanisms by which proteins are imported into the mitochondria, allowing for the expansion of the mitochondrial reticulum.

### 3.1. Fusion and Fission

Mitochondria are dynamic organelles that are continuously undergoing the processes of fusion and fission. Mitochondrial fusion is the expansion of the mitochondrial network that is accomplished by mitofusin 1 (Mfn1) and mitofusin 2 (Mfn2) in the outer mitochondrial membrane [143], as well as by optic atrophy 1 (OPA1) in the inner mitochondrial membrane [144]. These mitochondrial fusion proteins contain GTPase functional domains, which, when activated, lead to an expanded, elongated mitochondrial network. Mitochondrial fission is the process that opposes fusion, whereby the mitochondrial network can be divided, resulting in small, fragmented, and globular mitochondria. Fission is also governed by GTPase proteins such as dynamin-related protein (Drp1) and fission 1 protein (Fis1) [145, 146]. Healthy mitochondrial dynamics are regulated through the maintenance of a balance between these opposing processes, which is fundamental for sustaining mitochondrial quality and function in skeletal muscle. However, aging muscle appears to have an imbalance between the fusion and fission processes, thus disposing mitochondria toward undergoing either fusion or fission.

Several studies have revealed that aging skeletal muscle mitochondria may preferentially undergo fission, resulting in smaller and fragmented mitochondrial structures [8, 147]. For example, in a study comparing young (5 months) and old (35 months) Fisher 344 Brown Norway (BN) rats, aging skeletal muscles were observed to have elevated protein levels of Fis1 and Drp1, as well as downregulated Mfn2 levels, compared to their young counterparts [8]. Notably, a remarkable reduction in Mfn2 protein levels was also found in the skeletal muscle of old mice, and the age-related decline was shown to be progressive throughout aging [148]. This Mfn2 deficiency in aging muscle is also linked to mitochondrial dysfunction, along with diminished oxidative capacity [148], and this can contribute to muscle atrophy and weakness. Thus, the absence of Mfn2 may play a significant role in contributing to mitochondrial fragmentation and associated sarcopenia in aging muscle.

Contradictory results have been also reported that skeletal muscle may be more dependent on mitochondrial fusion in response to aging. Using a two-dimensional microscopic analysis, Leduc-Gaudet et al. showed more elongated SS mitochondria in the skeletal muscle of old mice, as well as more branched IMF mitochondria, as compared to those of young mice [9]. In this study, although no significant difference in fusion and fission proteins was found, the ratio of Mfn2 to Drp1 appeared to increase, indicating an elevation in the fusion index in the aging muscle [9]. It may be that mitochondrial fusion is more active than fission in the early-aging skeletal muscle of mice (~15 months old), as indicated by significantly increased Mfn1 and Mfn2 protein levels in the skeletal muscle, as well as decreased Fis1 protein levels [149]. In addition, other studies have revealed that both fusion and fission proteins are not changed [150] or are all upregulated [151] in the skeletal muscle of old animals. These inconsistent results in the process of mitochondrial dynamics may be due to differences in age, species, and/or muscle types of animals. Taken together, it is still unclear how aged skeletal muscle regulates fusion and fission to meet the aging-related alterations in mitochondrial structure and capacity.

### 3.2. Autophagy/Mitophagy

Autophagy is a “self-eating” system by which damaged organelles and cellular byproducts are degraded in the lysosome to help maintain cellular homeostasis. Autophagic substrates are nonselectively encapsulated by a double-membrane structure, an autophagosome, wherein they are conjugated and ubiquitinated by both the lipidated, active form of microtubule-associated protein 1A/1B-light chain 3 II (LC3-II) and p62. The autophagosome is then fused with a lysosome, whereupon an autolysosome is formed. The engulfed substrates are subsequently degraded by a variety of pH-dependent lysosomal proteases. Mitophagy is a mitochondria-specific form of autophagy. Damaged and/or dysfunctional mitochondria, characterized by a loss of mitochondrial membrane potential, recruit PTEN-induced kinase 1 (PINK1), which in turn activates parkin and leads to the ubiquitination of the outer membrane proteins. This mitochondria-ubiquitinated complex is encapsulated by autophagosome and then is degraded in the lysosome. Mitophagy has been identified to be crucial in maintaining healthy mitochondria in various tissues and disease states through the deletion of malfunctioning mitochondrial segments within the network.

The literature is replete with statements that the autophagy system is dysregulated with age [152–154], including within aged skeletal muscle [155, 156]; however, variations and contradictory findings are present in the literature [149, 155–157]. For instance, an increased accumulation of the autophagy markers p62 and LC3-II was observed in both slow (soleus) and fast (tibialis anterior) skeletal muscles of aged Fisher 344 BN rats [155], which may be indicative of diminished autophagic degradation in aging muscle, because LC3-II is degraded during autophagy. Others have shown that basal autophagic regulation in skeletal muscle may be not altered with aging [150, 158]. For example, muscle protein abundance of key autophagy markers such as Beclin-1, ULK1, and p62, as well as the protein ratio of LC3-II to LC3-I, is not different between young and older subjects [150, 158]. Meanwhile, the LC3 ratio (II to I) was observed to be lower in the skeletal muscle of middle-aged animals [159], suggesting that autophagy may be differentially regulated with age. Importantly, many of these observations are based on data in which autophagy (or mitophagy) flux has been not assessed. Colchicine, an inhibitor of autophagosome transport, is an effective chemical for estimating “autophagic flux.” Using colchicine, Baehr et al. provided some information to suggest that autophagy flux is impaired in the skeletal muscle of old animals [155]. However, much more research is required to clarify the contradictory findings in the literatures.

Recent studies have also sought to understand the effects of aging on mitophagy in skeletal muscle [160], even though the number of studies is limited. In *Drosophila*, Rana et al. have suggested the necessity of parkin not only for prolonging lifespan but also for sustaining mitochondrial quality and function in aging flight muscles [160]. They further suggest a notable link between parkin and mitochondrial fusion, as parkin overexpression appears to downregulate aging-related increases in Mfn abundance, thus accelerating the degradation of polyubiquitinated proteins and relieving mitochondrial proteotoxicity [160]. In contrast, a recent study by Sebastian et al. showed that the aging-associated Mfn2 deficiency may contribute to a decrease in mitophagic flux, as suggested by an increased accumulation of LC3-II and parkin on the mitochondria [148]. This aging-associated decline in muscle mitophagy seems to be compensated by increasing other signaling pathways including hypoxia-inducible factor 1-alpha (HIF1*α*) and BCL2/adenovirus E1B 19 kDa protein-interacting protein 3 (BNIP3) in a ROS-dependent manner [148].

AMPK is a key player in autophagy and mitophagy during starvation and aging [149, 161], and its activation in skeletal muscle appears to be diminished by aging [14, 149]. Bujak et al. showed that AMPK is important in maintaining mitochondrial integrity and mitophagic capacity in aging skeletal muscle [161]. In response to muscle-specific AMPK deletion, both SS and IMF mitochondrial sizes were shown to be increased in comparison to those of age-matched wild-type mice, along with a significant decline in mtDNA copy numbers [161]. In this animal model, remarkable accumulations of p62 and parkin proteins were also observed, thus indicating a link between AMPK and mitophagy in aging muscle [161]. In addition, Fritzen et al. also revealed data that following AMPK knockdown, the ratio of LC3-II to LC3-I was increased in the skeletal muscle of old mice as compared to that of age-matched animals [157]. AMPK has also been observed to regulate transcription factor EB (TFEB), a master regulator of lysosomal biogenesis. In mouse liver, AMPK is activated in response to starvation, which leads to the upregulation of autophagy and lysosomal genes via the interaction between TFEB and its coactivator, arginine methyltransferase 1 (CARM1) [162]. Whether this occurs in skeletal muscle is not known and further investigation is warranted.

Autophagy that is achieved via the direct transport of substrates into the lysosome is termed chaperone-mediated autophagy (CMA). This process requires carrier proteins such as heat shock cognate 70 (HSC70) to deliver the substrates to lysosomal-associated membrane protein 2A (LAMP2A), whereupon the substrates are translocated into the lysosomal lumen for degradation. In the context of the lysosomal system itself, aging may downregulate lysosomal activities, as LAMP2A [163] and HSC70 protein levels [164] are all reduced in the liver of old rats. To our knowledge, very few studies have been done to understand CMA in aging muscle, and a single study showed that HSC70 protein abundance appears to be elevated in the skeletal muscle of old (30 months) mice than in young (12 months) animals [165]. Moreover, the skeletal muscle of old Fisher 344 BN rats appeared to be characterized by lipofuscin accumulation within the lysosomal lumen [166], suggesting that defects in lysosomal function exist in aging muscle. It was also reported that LAMP2 mRNA levels are decreased in the aging plantaris of Fisher 344 BN rats as compared to those in young animals [167], which is supported by data revealing that the activity of the lysosomal protease cathepsin L was also lower in aging skeletal muscle, regardless of muscle type [155]. Thus, dysregulation of the lysosomal system may play a limiting role in aging muscle autophagic regulation. However, more detailed studies are needed to clarify the underlying mechanisms of CMA and other lysosomal activities in aging skeletal muscle.

### 3.3. Protein Import

Mitochondrial biogenesis is dependent on protein components encoded by both mitochondrial and nuclear DNA (mtDNA and nDNA, resp.). Only thirteen proteins are encoded by mtDNA, while the remainder (~1100) are dependent on the transcription of nDNA. Proteins that are encoded by nDNA are transported from the cytosol to the mitochondrial membrane import machineries, named the translocases of the outer/inner membrane (TOM/TIM). Through these complexes, the proteins are moved into mitochondrial matrix by the mitochondrial heat shock protein 70 (mHSP70) and are subsequently posttranslationally modified by mitochondrial processing peptidase (MPP). This important machinery contributes importantly to the management of mitochondrial quality and the correct stoichiometry of ETC components in skeletal muscle mitochondria.

Although it was shown that TOM proteins (e.g., TOM22) are not changed in the aging skeletal muscle of humans [147] and rodents [149], studies using mitochondrial fractions have suggested that the aging process may lead to the upregulation of TOM protein levels in skeletal muscle [168]. For example, key protein markers for TOM complex such as TOM40 and TOM22 were increased in the muscle mitochondrial fraction of old rats, as compared to those in young animals [168]. Moreover, it was also shown that TOM22 levels are increased in the skeletal muscle of functionally inactive old subjects [147]. Taken together, it seems likely that the mitochondrial protein import system is activated in order to compensate for age-related mitochondrial dysfunctions in skeletal muscle.

## 4. Exercise, Mitochondrial Adaptations, and Aging

**Figure 2:**
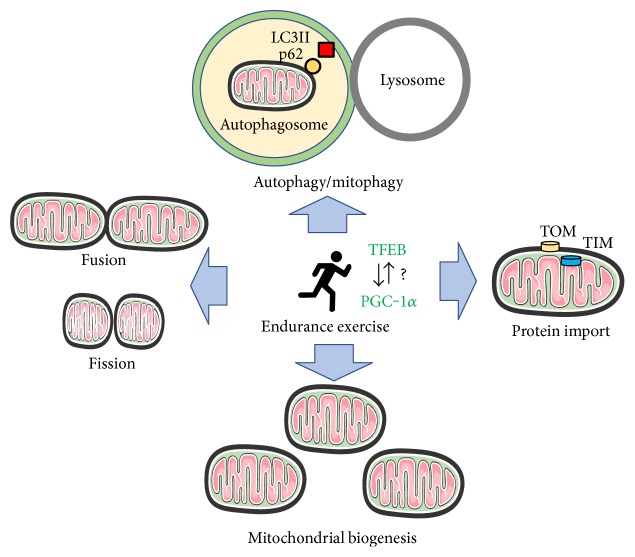
Exercise and mitochondrial dynamics in aging muscle. Endurance exercise training increases mitochondrial biogenesis in aging muscle, although its extent may be lessened compared to young muscle. In addition, chronic exercise leads to a global upregulation of protein markers for mitochondrial dynamic controls: fusion/fission, autophagy/mitophagy, and protein import. Since the lysosomal system has been suggested as a key player for governing mitochondrial quality control, the role of TFEB, a master regulator of lysosomal biogenesis, appears to be important and its relationship with PGC-1*α* may be also considerable for the exercise-inducible upregulation of mitochondrial turnovers. However, more studies are needed to clarify the effects of endurance training exercise on the mitochondrial turnover systems in aging muscle.

Exercise has been relatively well accepted as an effective strategy for delaying either the onset or the progression of sarcopenia; however, its effects on mitochondrial biogenesis and turnover have been less studied in aging skeletal muscle. In the following sections, we outline studies focusing on the effects of exercise on mitochondrial quality control in aging skeletal muscle (Figure 2).

### 4.1. Exercise and Mitochondrial Biogenesis

As compared to young, healthy skeletal muscle, fewer studies have been accomplished to delineate the effects of exercise on mitochondrial quality controls in aging skeletal muscle. Endurance exercise training or chronic contractile activity (CCA) successfully leads to mitochondrial adaptations in aging skeletal muscle, to a lesser extent than which is observed in young muscle [51, 151, 169]. Following endurance training, aging skeletal muscle does exhibit increases in the gene and protein abundances of PGC-1*α*, accompanied by increases in mtDNA, mitochondrial mass, ETC components, and mitochondrial transcriptional regulators such as Tfam [151, 169]. These training-inducible mitochondrial adaptations are regulated by various signaling pathways. Several studies have reported that endurance training activates AMPK, p38 mitogen-activated protein kinase (MAPK), and SIRT1 in the skeletal muscle of old rodents and humans, all of which are activators of PGC-1*α* and thus of mitochondrial biogenesis [151, 169]. Nonetheless, some findings have suggested that the decline in mitochondrial markers was not prevented in the skeletal muscles of animals at advanced age (i.e., 34~36-month-old Fisher 344 BN rats) in response to endurance training [14, 170]. Also, it was reported that mitochondrial biogenesis following 12 weeks of cycling exercise training was attained in the skeletal muscle of old women without an increase in PGC-1*α* protein [171], suggesting possible alternative signaling pathways to lead to exercise-induced mitochondrial biogenesis in aging muscle, as compared to young muscle [172].

Research trials employing lifelong physical activity also suggest an important role of chronic muscle activity in the maintenance and improvement in mitochondrial integrity and aerobic performance that are attenuated with aging [173, 174]. Higher mitochondrial volume density following lifelong exercise is observed, and it is highly correlated with aerobic capacity (VO_2max_) in the skeletal muscle of healthy individuals over 60 years old [175].

Resistance exercise for aging individuals has been well defined as an effective regimen for lessening aging-associated muscle atrophy and weakness [176, 177]. However, only few studies have sought to investigate the underlying mechanism by which resistance exercise alters mitochondrial abundance and function in aging skeletal muscle. Acute resistance exercise with leg extensions was shown to significantly increase the mRNA levels of both total PGC-1*α* and PGC-1*α*4, as well as Tfam, in the muscle of aged men [178]. While Flack et al. [179] observed no changes in mitochondrial markers in the skeletal muscle of individuals over 60 years old following 12 weeks of resistance exercise, other studies have shown that 6 months of resistance training partially reversed the aging-related dysregulation of genes for mitochondrial function [176]. Furthermore, a mixed type of chronic exercise (voluntary resistance wheel exercise training) was found to have a significant effect on increasing muscle aerobic capacity in aging muscle, as well as muscle mass and size [158], indicating the therapeutic potential of using a mixed training type to prevent the atrophy and reduction in mitochondria that is associated with age.

### 4.2. Exercise and Mitochondrial Turnover

#### 4.2.1. Fusion/Fission and Exercise

Recent studies have sought to understand the effects of exercise on mitochondrial dynamics in aging skeletal muscle. While mixed results have been found in young skeletal muscle [180, 181], several studies have shown parallel changes in both fusion and fission proteins in aging skeletal muscle following chronic physical activity or endurance exercise training [151, 182, 183]. For example, 6 weeks of treadmill exercise upregulated both Fis1 and Mfn1 protein abundances in the skeletal muscle of old animals [151]. Furthermore, lifelong physically active older women demonstrated elevated levels of both Mfn2 and Drp1 mRNA in their skeletal muscle as compared to age-matched inactive women [184]. Hence, chronic muscle activity seems to control mitochondrial dynamics in aging skeletal muscle through the coregulation of both fusion and fission processes.

#### 4.2.2. Autophagy/Mitophagy and Exercise

Although contradictory findings have been observed [185, 186], acute aerobic exercise is likely to increase the autophagic responses in skeletal muscle [187]. For example, a bout of treadmill exercise elevated muscle and mitochondrial autophagic flux in young skeletal muscle, wherein LC3-II and p62 fluxes were upregulated immediately after exercise, as well as during recovery [187]. Interestingly, this acute exercise-related increase in muscle autophagic and mitophagic flux appeared to be diminished in the absence of PGC-1*α*, which suggests the importance of PGC-1*α* on the exercise-inducible muscle remodeling [187]. This is also supported by a study by Vainshtein et al. [188], wherein denervation-induced upregulation of the mitophagy system was lessened in the skeletal muscle of PGC-1*α*-KO mice. Future work will be required to determine whether PGC-1*α* plays a key role inactivity-dependent changes in autophagy/mitophagy in aging skeletal muscle.

Several studies have sought to understand endurance training effects on the cellular systems in aging muscle. For instance, it has been claimed that endurance exercise training may upregulate the autophagy process in the skeletal muscle of old animals [158, 189, 190]. However, these interpretations have been derived from measurements of the ratio of LC3-II to LC3-I without changes in p62 protein accumulation [158], which is a limitation as they did not measure autophagy flux. Nonetheless, in response to endurance training, these aging muscles were shown to have increased expression of autophagic markers such as autophagy-related protein 7 (ATG7) and Beclin-1 that are all significant players in the formation of the autophagosome. The CMA protein LAMP2A also followed the same pattern [185]. Further, physically active elderly individuals have increased mRNA levels of autophagy markers such as Beclin-1, ATG7, and p62 [173, 184], and they also had increased mitophagy markers including BNIP3 and parkin in the muscle [173, 184]. Indeed, in a recent study (in Press), we observed that muscle autophagy may be concomitantly altered along with mitochondrial adaptations over the course of chronic muscle activity. In addition, lysosomal proteins appear to adapt prior to mitochondrial changes. Therefore, it is possible that endurance exercise training or chronic muscle activity may lead to a mitochondrial remodeling in the skeletal muscle of aged individuals, but more studies are warranted to clearly understand endurance exercise training effects on the autophagy/mitophagy systems.

Autophagic responses following resistance exercise are shown to be different from the endurance exercise-induced changes discussed above. For example, it has been reported that in response to a bout of resistance exercise, the protein ratio of LC3-II to LC3-I was decreased in the muscle of old individuals compared to that in the young group [191, 192], while p62 protein accumulated [178]. As in the acute responses, resistance training appears to accelerate autophagic degradation (flux) in aging skeletal muscle. For example, 6 weeks of ladder climbing exercise training was shown to downregulate the ratio of LC3-II to LC3-I and p62 protein abundance in the muscle of old rats [193]. In this study, other autophagy protein markers including Beclin-1, ATG7, and cathepsin L were all upregulated in the aging muscle [193], collectively suggesting that resistance exercise may accelerate autophagy with age.

#### 4.2.3. Protein Import and Exercise

As in other aging studies, there have been few studies examining the effects of exercise or chronic activity on the mitochondrial protein import system in aging muscle. Using a CCA model, Ljubicic and Hood [14] observed an attenuated change in protein import systems (TIM17, TIM23, and mtHSP70) in aging skeletal muscle following 7 days of CCA, whereas the same markers were significantly elevated by CCA in young muscle. In addition, Joseph and colleagues have shown that the increase in protein import with CCA is correspondingly reduced in aged muscle [168]. Thus, while the import process is not affected with age basally, the adaptive potential in response to exercise appears to be reduced.

## 5. Conclusion

Mitochondrial quality control in aging skeletal muscle is regulated via mitochondrial biogenesis and mitochondrial turnover; however, the regulation of these processes seems to be less sensitive to the effects of exercise compared to that in young, healthy muscle. Regulation of mitochondrial quality in skeletal muscle can be also accomplished by other cellular systems including ubiquitin proteasomal degradation, lysosomal regulation, and apoptosis. In particular, the lysosomal system has been recently suggested as a key player for regulating autophagy/mitophagy, as well as mitochondrial energy balance [194]. Indeed, a key component of lysosomal biogenesis, the transcription factor TFEB, appears to determine exercise capacity [194], and we have suggested a coordinated function between TFEB and PGC-1*α* during both denervation- [188] and CCA- (in Press) induced skeletal muscle remodeling, suggesting an importance of maintaining a balance between mitochondrial biogenesis and lysosomal system for the muscle quality control. Therefore, it will be interesting for future studies to examine aging-related alterations in the lysosomal system in skeletal muscle, as well as to study how endurance and/or resistance exercise regulates lysosomal capacity in aging muscle. These findings will suggest a possible pharmaceutical target for improving aging-related mitochondrial dysregulation in skeletal muscle.

It is evident that maintaining healthy mitochondrial quality is essential for defeating aging-related muscle dysfunction and weakness. To better understand the regulation of mitochondrial quality control in aging muscle, more studies are warranted to reveal the underlying mechanisms behind the effects of exercise on the mitochondrial biogenesis and turnover. Hopefully, the results will suggest the most effective exercise strategies for attaining optimal mitochondrial quality in aging skeletal muscle.

## Abbreviations

AICAR: 5-Aminoimidizole-4-carboxamide ribonucleotide
AMP: Adenosine monophosphate
AMPK: AMP-activated protein kinase
ATG7: Autophagy-related protein 7
ATP: Adenosine triphosphate
BNIP3: BCL2/adenovirus E1B 19 kDa protein-interacting protein 3
CARM1: Coactivator-associated arginine methyltransferase 1
CCA: Chronic contractile activity
CMA: Chaperone-mediated autophagy
COX: Cytochrome c oxidase
DNA: Deoxyribonucleic acid
Drp1: Dynamin-related protein 1
ERR: Estrogen-related receptor
ETC: Electron transport chain
Fis1: Fission 1 protein
Fisher 344 BN rats: Fisher 344 Brown Norway rats
FoxO3a: Forkhead box O3a
GTP: Guanosine triphosphate
HIF1*α:*: Hypoxia-inducible factor 1-alpha
HSC70: Heat shock cognate 70
IMF: Intermyofibrillar
KO: Knockout
LAMP2A: Lysosomal-associated membrane protein 2A
LC3-I or -II: Microtubule-associated protein 1A/1B-light chain 3 I or II
MAPK: Mitogen-activated protein kinase
Mfn1/2: Mitofusins 1 and 2
mHSP70: Mitochondrial heat shock protein 70
MPP: Mitochondrial processing peptidase
mtDNA: Mitochondrial DNA
TFB1/2: Transcription factors B1 and B2
NAD^+:^: Nicotinamide adenine dinucleotide
nDNA: Nuclear DNA
NMN: Nicotinamide mononucleotide
NMNAT: NMN adenylyltransferase
NRF-1/2: Nuclear respiratory factors 1 and 2
NUGEMP: Nuclear genes encoding mitochondrial proteins
OPA1: Optic atrophy 1
OXPHOS: Oxidative phosphorylation
p53: Tumor protein 53
p62: Ubiquitin-binding protein p62
PGC-1*α:*: PPAR-coactivator 1 alpha
PINK-1: PTEN-induced kinase 1
POLRMT: Mitochondrial RNA polymerase
PPAR: Peroxisome proliferator-activated receptor
ROS: Reactive oxygen species
rRNA: Ribosomal ribonucleic acid
SIRT1: Silent mating type information regulation 2 homolog 1
SS: Subsarcolemmal
Tfam: Transcription factor A of the mitochondria
TFEB: Transcription factor EB
TIM: Translocase of the inner mitochondrial membrane
TOM: Translocase of the outer mitochondrial membrane
tRNA: Transfer RNA.
